# The Skin Ivory Spot. A Possible Indicator for Skinfield Photo-Carcinogenesis in Recreational Sunbed Addicts

**DOI:** 10.3390/ijerph9020362

**Published:** 2012-01-25

**Authors:** Pascale Quatresooz, Claudine Piérard-Franchimont, Gérald E. Piérard

**Affiliations:** 1 Laboratory of Skin Bioengineering and Imaging, Department of Dermatopathology, University Hospital of Liège, Belgium; 2 Department of Dermatology, Regional Hospital of Huy, Belgium; 3 University of Franche-Comté, Hôpital Saint-Jacques, Besançon, France

**Keywords:** carcinoma, melanin, melanocyte, ULEV method, ultraviolet light, sunbed, field photocarcinogenesis

## Abstract

*Introduction*: For a decade or so, artificial sources of restricted light wavelengths, particularly sunbeds, have progressively gained popularity among adolescents and young adults. Warnings were raised focusing on the risk of accelerated photoaging and photocarcinogenesis. The ULEV (ultraviolet light-enhanced visualization) method is a convenient noninvasive way identifying subtle pigmentary changes presenting as a mottled subclinical melanoderma (MSM). Of note, rare spotty amelanotic macules presenting as skin ivory spots (SIS) was reported on any part of the body. *Subjects and method*: This work is the first attempt at evaluating the changes in the MSM and SIS spots developed on the skin of 33 phototype III young women designated as avid users involved in frequent exposures to sunshine and sunbeds for lifestyle purposes for a duration of at least 120 months. *Results*: MSM was markedly heterogeneous and was distinctly obvious in the majority of adepts of frequent natural and artificial photoexposures. SIS was particularly developed in subjects presenting with severe MSM patterns. *Discussion*: MSM and SIS are more severe in subjects frequently exposed to sunbeds and sun exposures. These signs possibly represent a risk marker for field photocarcinogenesis.

## 1. Introduction

Photoaging results from chronic exposures to non-ionizing electromagnetic radiation. Early clinical signs are recognized by a mottled (mosaic) subclinical melanoderma (MSM) [1,2]. Loss of skin firmness, solar elastosis and coarse wrinkles develop later. These varied clinical changes are primarily due to chronic actinic radiations. Additional exposures to artificial sources of restricted light wavelengths are increasingly being used for lifestyle purposes in many affluent cultural societies [3]. In general, tanning benches are problematic, particularly in conditions of unsupervised and non-medical use [3,4,5,6,7,8,9,10].

Among the light spectrum reaching the Earth’s surface, the relevant wavelength spectrum regarding photoaging encompasses UVA (λ = 320-400 nm) and UVB (λ = 280-320 nm), as well as infrared A (IRA, λ = 760-1440 nm) and visible light (λ = 400-760 nm). Ultraviolet (UV) A represents the bulk of the radiant energy emitted by commercial sunbeds. There is evidence that these radiations induce a variety of skin cancers, including nonmelanoma skin cancers (NMSC) and cutaneous malignant melanomas (CMM) as well [11]. The amount of radiation in the UVB spectrum is usually low in sunbeds, but “fast tanning” lamps emit a higher proportion of UVB. The additional effects of infrared (IR) radiations emitted by sunbeds should not be dismissed [12,13].

The acquired discrete uneven skin pigmentation forming the MSM patterns is a hallmark of photoaging [1,2,14]. Once delivered by melanocytes to keratinocytes, melanin acts in part as a UV-filter [15]. However, according to individual parameters including the phototype, age and cumulative UV light exposure, skin presents distinct MSM appearances [2]. The diverse patterns of age-related MSM is conveniently disclosed and magnified using the computerized UV light-enhanced visualization (ULEV) method [1,2,14]. A CCD camera equipped with an internal UV-emitting unit is suited for such precise quantification of the epidermal melanin content. The increased contrast between the faint MSM and the near skin is the combined result of: (a) the greater reflection of UV wavelengths than visible light by dermal collagen and (b) the greater UV absorption by epidermal melanin. Any other UV-absorbing or reflecting structure interposed above the collagen network alter the aspect at the ULEV examination [2]. Previous observations indicated that some individuals exhibited rare foci of nearly total depigmentation [2,16]. The focal amelanotic skin appeared as white irridescent skin ivory spots (SIS).

The present study was conducted in order to scrutinize some effects of skin tanning in young women enthusiasts of sun parlors. Specific field distributions of MSM and SIS were searched over the head and limbs.

## 2. Subjects and Methods

The present observational study was approved by the University Hospital Ethics Committee (B70720084873) and performed in accordance with the Declaration of Helsinki. The noninvasive observations were conducted with the understanding and consent of the volunteers who had been previously involved in similar studies [5,8]. Thirty-three phototype III women aged 30-34 years were selected as test subjects (TS). They were lifelong residents of the Liège region, and they were avid sunbed and sunshine users. For at least 120 months, they used commercial tanning facilities on a regular basis in order to maintain a deep tan. Sunbeds were outfitted with non-prescription lamps. Irradiance measurements were not performed. None of the volunteers were taking photoactive medications. A gender, age and phototype-matched group avoiding excessive UV (nature and sunbed) exposures served as control subjects (CS). No other possible confounding factors, such as sun protection habits, were presently considered.

ULEV was performed using a computer-assisted CCD camera equipped with an internal UV-emitting unit (Visioscan® VC98, C+K electronic, Cologne, Germany). The camera was closely applied to the skin surface of the forehead, temples, the dominant dorsal forearm and the homolateral anterior aspect of a leg. At each site, the MSM aspect was recorded on three contiguous fields (8*10 mm). The generated digital signal corresponded to 256 gray levels, ranging from zero for black to 256 for white. The examined sites were clinically featureless without any obvious melanin pigmentation heterogeneity, and without any telangiectasia or other vascular hyperplasia. The descriptive MSM patterns were assessed as described in Table 1. The relative area (%) of the darker spots was assessed using image analysis (MOP-Videoplan, Kontron, Eching, Germany) averaged for the three examination fields. In addition, areas of intense brightness suggesting an almost absence of melanin in SIS was also searched for.

**Table 1:** Patterns of mottled subclinical melanoderma according to [1].

Pin-point	Minute irregularly distributed darker spots
Follicular dots	Speckled perifollicular darker rings
Small macules	Small interfollicular darker areas
Globular macules	Accretive and circinate confluence of smaller macules
Streaky macules	Elongated darker areas along wrinkles
Confluent macules	Massive darker areas

Data were expressed as means ± standard deviations (SD) for the relative area and as proportions of the predominant patterns on each assessed site. The density function of the relative area was estimated by a non-parametric fitting method. The comparison of the distribution of patterns between TS and CS was performed by the chi-square test, while mean areas were compared by the Student’s t-test. Results were considered to be significant at the 5% critical level (p < 0.05). All calculations were performed using SAS (version 8.2 for Windows) and S-PLUS (version 6.1) statistical packages.

## 3. Results

Compared to CS the MSM aspect in UV-addict TS was significantly (p < 0.04) altered, showing an increase in the relative area of darker spots. Under closer examination, three grades of MSM changes were disclosed. The first MSM grade (TS: 31/132, CS: 75/132) was characterized by relative MSM areas lower than 30%. The second MSM grade (TS: 58/132, CS: 39/132) was characterized by a relative area of the darker MSM spots ranging 31-60%. The third MSM grade (TS: 43/132, CS: 18/132) was characterized by a value above 60% in the extent of the darker MSM area. The MSM patterns were evenly distributed over the whole scrutinized areas. They corresponded to tiny areas a few millimetres across, which differed sharply from the contiguous skin in one or more respects. This aspect was not visible under regular examination at white light illumination. The larger spots were irregularly shaped, and spaced out. Their sizes were uneven, occupying several square millimetres.

**Figure 1:**
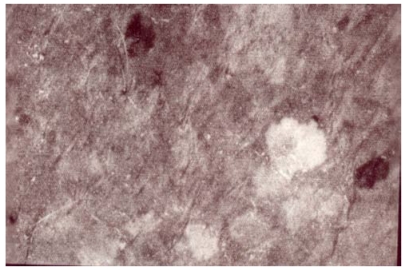
A woman’s leg. Single SIS in a patchwork of spotty areas of MSM of distinct darkness.

**Figure 2:**
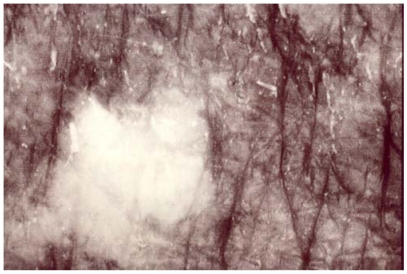
A women’s forehead. A sharply circumscribed medium-size SIS.

**Figure 3:**
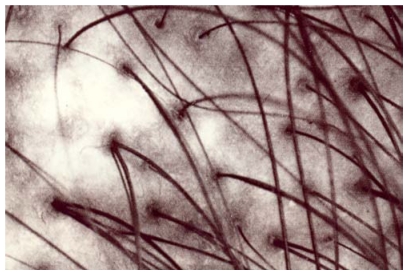
A man’s occipital area. A larger fuzzy amelanotic area.

The melanotic spots MSM were admixed with a few amelanotic SIS scattered among the MSM structures. Annular and circinate figures of MSM and SIS were never disclosed. The SIS were iridescent and uniform in color. Their sizes were variable (Figure 1, Figure 2, Figure 3). They were never clustered. A total of 17/132 SIS were disclosed on any body site of TS, but they predominated over the dorsal forearms and on the anterior aspect of the legs. By contrast, only 2/132 SIS were disclosed in CS. Hence, the melanin mapping over the light-exposed limb areas combined both hyper- and hypomelanotic spotty areas. 

## 4. Discussion

The present long-term study was an attempt at identifying some amelanotic SIS changes appearing in frequent users of tanning salons. The volunteers were avid UV-users and they attended the tanning facilities all the year around. This work is not a case-control study because the number, duration and irradiance of sun and sunbed exposures were not controlled. Indeed, the findings represent the combination effects of natural and artificial tanning sources. The effect of the possible application of sun protectors was not presently considered.

The Visioscan® VC98 examination was influenced by the melanin filtration of both the incident UV light and the reflected fluorescent light from the UV-excited dermal fibrous network. The incident light was in part reflected and scattered at the skin surface, corresponding to the specular light reflectance [16]. Another part of the incident light penetrated the epidermis where it was in part absorbed by melanin. The residual incident UV light was reflected as fluorescence by the dermal fibrous matrix that in turn was filtered again by the epidermal melanin load during its way back. Doubtless, SIS followed MSM in their occurrence, and the amelanotic spots should be regarded as a severity factor of actinodermatosis.

The frequency with which epidermal cells misbehave in circumscribed groups is apparently influenced by chronic UV exposures. The present study points to: (a) the melanotic changes in young adults and (b) to the possible combined effect of natural exposure during sunny days and artificial exposure in recreational sunbeds. It is unknown how, when and why the melanocyte-keratinocyte interactions form circumscribed groups of cells sharing a common biologic activation. The sharp circumscription of each MSM and SIS does not necessarily correspond to the boundary between one “clone” of melanocytes and the rest, but it possibly represents the margin of a field of influence by the inducing factor. The reason for the sharp spread limitation by the field of abnormality is unsettled. Similarly, the actual mechanism responsible for spotty field changes is not yet disclosed. The putative nature of any pathogenic event following UV exposure which could be held responsible for inducing the abnormal functional behavior of previously normal cells, remains at present entirely speculative.

On the long term, MSM was shown to be possibly smoothed by sun protection, and it was partly reversed by topical applications of calcipotriol [17]. No information is yet available about the biological consequences of alleviating or smoothing MSM. However, a statistical correlation was previously established between MSM severity on the face and the occurrence of multiple basal cell carcinomas [2]. It has been hypothesized that photo-induced NMSC possibly developed from the amelanotic SIS because the natural photoprotection had been abolished at these sites. The decline in the TS skin condition appeared unusually rapid compared with CS.

Any relationship between MSM, SIS and CMM was not explored so far. However, the reported increased incidence of CMM in young adults adept of sunbeds [18,19] now invite exploring the predictive value of MSM and SIS in CMM. This might be of particular interest in teenagers and young adults [6,7,20,21,22,23,24]. The same method is conveniently offered for distinguishing intrinsic aging from photoaging [25,26,27]. Of note, all MSM patterns are not associated with a global aging process. For instance, the so-called speckled perifollicular melanosis of the face and scalp develops during early adolescence and is probably influenced by hormonal activation [14,28].

## 5. Conclusions

The out-and-out skin tanning addiction in some segments of the population [29] is at risk for photoaging and photocancerogenesis. The ULEV method is offered as a tool for identifying subjects susceptible to progress in the relevant biologic degradation. Further prospective studies are necessary to explore the links of MSM and SIS with field photocarcinogenesis.
